# Full-Length Transcriptome of the Great Himalayan Leaf-Nosed Bats (*Hipposideros armiger*) Optimized Genome Annotation and Revealed the Expression of Novel Genes

**DOI:** 10.3390/ijms24054937

**Published:** 2023-03-03

**Authors:** Mingyue Bao, Xue Wang, Ruyi Sun, Zhiqiang Wang, Jiqian Li, Tinglei Jiang, Aiqing Lin, Hui Wang, Jiang Feng

**Affiliations:** 1 College of Life Science, Jilin Agricultural University, Changchun 130118, China; 2 Jilin Provincial Key Laboratory of Animal Resource Conservation and Utilization, Northeast Normal University, Changchun 130117, China

**Keywords:** *Hipposideros armiger*, full-length transcriptome, PacBio, genome annotation, SMRT sequencing

## Abstract

The Great Himalayan Leaf-nosed bat (*Hipposideros armiger*) is one of the most representative species of all echolocating bats and is an ideal model for studying the echolocation system of bats. An incomplete reference genome and limited availability of full-length cDNAs have hindered the identification of alternatively spliced transcripts, which slowed down related basic studies on bats’ echolocation and evolution. In this study, we analyzed five organs from *H. armiger* for the first time using PacBio single-molecule real-time sequencing (SMRT). There were 120 GB of subreads generated, including 1,472,058 full-length non-chimeric (FLNC) sequences. A total of 34,611 alternative splicing (AS) events and 66,010 Alternative Polyadenylation (APA) sites were detected by transcriptome structural analysis. Moreover, a total of 110,611 isoforms were identified, consisting of 52% new isoforms of known genes and 5% of novel gene loci, as well as 2112 novel genes that have not been annotated before in the current reference genome of *H. armiger*. Furthermore, several key novel genes, including *Pol*, *RAS*, *NFKB1*, and *CAMK4*, were identified as being associated with nervous, signal transduction, and immune system processes, which may be involved in regulating the auditory nervous perception and immune system that helps bats to regulate in echolocation. In conclusion, the full-length transcriptome results optimized and replenished existing *H. armiger* genome annotation in multiple ways and offer advantages for newly discovered or previously unrecognized protein-coding genes and isoforms, which can be used as a reference resource.

## 1. Introduction

Great Himalayan Leaf-nosed bats, *Hipposideros armiger*, a typical CF-FM (Constant Frequency-Frequency Modulation) bat [1], belong to the genus Hipposideros in the Hipposideridae family of Yinpterochiroptera. They often exhibit Doppler shift compensation (DSC) when flying or hunting, recognize target objects, and capture prey effectively in complicated environments with developed echolocation capabilities [2]. *H. armiger* is one of the largest species in the family of Hipposideridae, with an average body weight of about 60 g. Adults have a dark tan body color and are widely distributed in the Old World’s ecologically diverse habitats of tropical, subtropical, and Southeast Asia. In China, they are found in Shaanxi, Sichuan, Anhui, Guangxi, and Guizhou [3,4]. *H. armiger* is a gregarious species with a polygamous mating system [5], usually living in large clusters in warm and humid caves, where they are active in deep cave habitats and frequently vocalize for social communication in addition to echolocation, with a minimum spacing of 10–15 cm between individuals [6,7,8]. Species of the Hipposideridae family are major natural enemies of pests that destroy crops, forests, and nurseries [9,10], which is an important part of the global ecosystem and plays a significant role in maintaining the ecosystem’s balance. They can also effectively control the reproduction of pests and maintain the ecological environment.

Researchers have studied *H. armiger* more extensively in recent years, mainly in terms of Doppler shift compensatory behavior and echolocation [1,2,3,8,11], epigenetics [12], phylogeography and population genetics [13], and whole mitochondrial genome [14]. Guo et al. [15] isolated eight microsatellite loci from an enrich genomic library of *H. armiger* and found that these microsatellite markers will facilitate the study of population genetics in *H. armiger* and other bat species. Xu et al. [4] evaluated the geographical patterns of *H. armiger* genetic structure in China by analyzing the sequence variation of mitochondrial DNA control regions. Using two mitochondrial DNA (mtDNA) regions and seven nuclear microsatellite loci (nSSRs) from 216 individuals of *H. armiger*, Lin et al. [16] reported the divergence and population expansion history of *H. armiger*. In 2017, Dong et al. [17] de novo assembled *H. armiger* using the Next-generation genome sequencing technology, which yielded a total of 476.5 Gb of sequence with a predicted genome size of 2.18 Gb. While studies related to revealing the molecular basis of its high-frequency hearing at the genetic level are still in the exploratory stage, many related studies at the molecular level now urgently require high-quality genomic data as support. Even if data from short-read long sequencing have been accumulated in recent years, as in the above article, and genome sequencing of *H. armiger* has been completed, they do not provide full-length (FL) sequences for each RNA, thus limiting their usefulness in defining alternative splicing. Currently, there is still insufficient information on Alternative Splicing (AS), Alternative Polyadenylation (APA) loci, lncRNA, and fusion transcripts. Moreover, in some cases, short-read sequencing can produce low-quality transcripts, which can lead to incorrect annotation. Therefore, the establishment of a complete and comprehensive genomic database is crucial for us to subsequently initiate in-depth studies on echolocating bats.

Despite the rapid development of short-read transcriptome sequencing technologies in recent years, there are still many limitations [18,19,20,21]. For instance, next-generation sequencing technologies cannot identify genes longer than 5000 bp in most cases, and it is not easy to identify alternative splicing events that span the entire transcriptome. Currently, Pacific Biosciences (PacBio) single molecule real-time (SMRT) sequencing technology provides high quality FL transcripts by Isoform Sequencing (IsoSeq). IsoSeq has significant advantages over RNA-seq in terms of generating FL transcripts. Its most significant feature is single-molecule real-time sequencing, where the sequencing process eliminates the need for PCR amplification. Good evidence was provided for transcription of each gene by producing longer reads without the need for assembly. For example, accurate gene length (maximum >2000 bp), identifiable alternative splicing transcripts, and easy screening of novel transcripts can often improve the accuracy of existing gene models [22,23,24], especially for non-model species with low quality genomes or without reference genomes [25]. Previous studies have shown that even in the most thoroughly studied human genomes, the large number of splice variants identified by IsoSeq data could not be covered by the current annotations [26]. Wang et al. (2016) [27] generated 111,151 transcripts from six tissues of maize by using SMRT sequencing to capture 70% of the genes annotated in the maize reference genome, optimizing the original genome annotation and identifying a large number of novel long non-coding RNAs (LncRNAs) and fusion transcripts. Therefore, taking use of IsoSeq to construct a complete set of transcriptional isoforms for non-model organisms is highly warranted.

Considering the advantages of the complete structure of full-length transcripts in improving genome structure annotation, herein we aim to improve the accuracy of gene models in the *H. armiger* genome by using novel protein-coding transcripts and LncRNAs obtained from full-length transcriptome sequencing, optimizing existing reference genome annotations to facilitate comparative and functional genomic studies of constant-frequency echolocation bat species and their relatives. It will also provide a valuable molecular resource and data base for subsequent studies of high-frequency auditory nerve function genes in echolocation bats, further exploration of adaptive evolutionary mechanisms, and future studies of functional genomics in *H. armiger*.

## 2. Results

### 2.1. PacBio Iso-Seq Sequencing Data Analysis

**Figure 1:**
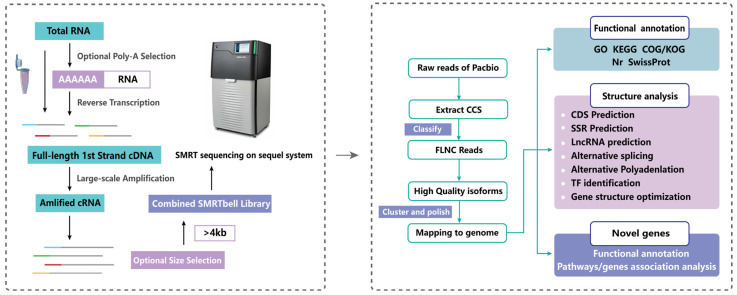
Sequel full length transcriptome cDNA library construction, data processing, and bioinformatic analysis.

**Figure 2:**
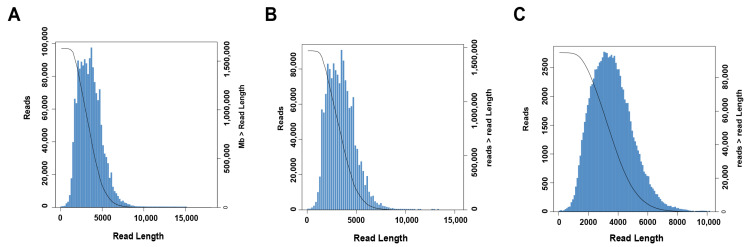
Length distributions of PacBio SMRT sequencing. (**A**) Number and length distributions of 1,630,747 CCS sequences. (**B**) Number and length distributions of 1,472,058 FLNC sequences. (**C**) Number and length distributions of 94,981 consistent sequence. The blue area is the number of reads at a certain length (corresponding to the left abscissa value), the black line corresponds to the ordinate on the right and is the number of reads greater than a certain length (corresponding to the right abscissa value).

According to the analysis flow chart shown in Figure 1, in order to obtain a representative full-length transcriptome of *H. armiger*, raw data generated by PacBio Sequel2 was analyzed using SMRT Link V8.0.0, and 40,724,724 subreads (120 Gb) were obtained from full-length sequencing, with an average length of 3082 bp and N50 of 3573 bp (Table 1). In order to provide accurate sequence information, the sequences with a number of full pass greater than or equal to 1 in the offline data were selected for circular consensus sequence (CCS) analysis. Finally, high-accuracy CCS reads (also known as HIFI reads) for subsequent transcript analysis were obtained. A total of 1,630,747 CCSs with an average length of 3535 bp were obtained (Figure 2A, Table 1), which were used to identify full-length nonchimeric reads (FLNC). Lima software (version 1.7.0) and IsoSeq refined software (version 3.0) were used to identify 5′/3′ primer and polyA constructs and chimeras, respectively. Finally, the above two data were merged, and the linker, polyA, and chimeric sequences were removed to obtain full-length non-chimeric sequences (FLNC reads), which were the original full-length transcript sequences of the species. A total of 1,472,058 FLNC reads with an average length of 3440 bp were obtained (Figure 2B, Table 1).

Next, we obtained unpolished consensus isoforms by hierarchically clustering similar FLNC reads (Figure 2C, Table 1). A total of 94,981 CCSs were obtained using the Quiver algorithm, including 3504 low-quality isoforms and 91,477 high-quality isoforms, with high-quality isoforms accounting for 96.31% of the total number of isoforms.

### 2.2. Mapping to the Reference Genome and Identification of Novel Isoforms

**Figure 3:**
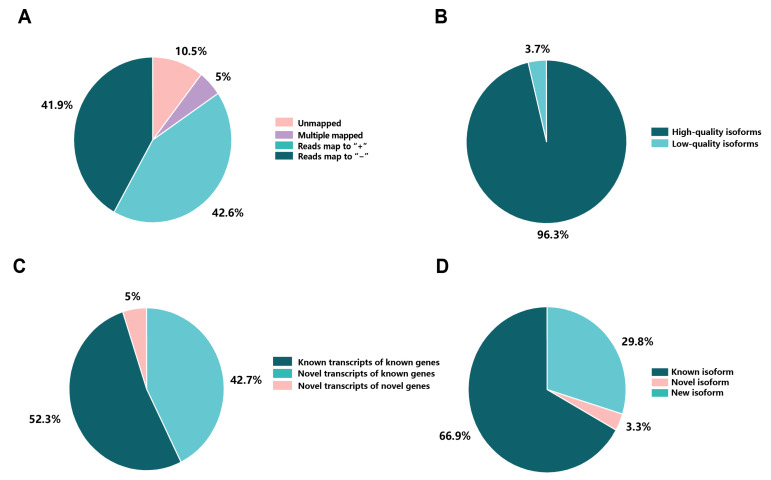
Pie charts of gene expression. (**A**) Statistical results of consistency sequence classification quantity. (**B**) GMAP analysis of corrected reads to reference genome. (**C**) Classification of transcript isoforms identified. (**D**) Statistics of gene expression results.

The 91,477 high-quality (HQ) isoforms were mapped to the reference genome of *H. armiger* (ncbi_GCF_001890085.1). Based on the mapping results, these reads can be classified into four groups: Unmapped, Multiple mapped, Reads map to “+”, and Reads map to “−”. A total of 81,871 (89.50%) reads were mapped to the reference genome, including 77,214 (84.41%) uniquely mapped reads and 4657 (5.09%) multiple mapped reads. A total of 38,948 (42.58%) reads were mapped to Reads map to ‘+’ and 38,266 (41.83%) reads were mapped to Reads map to ‘−’ (Figure 3A, Table S1). For the subsequent analysis, we only used HQ isoforms (Figure 3B, Table S1).

After correction, 110,611 transcripts were obtained, which were divided into three groups (Figure 3C, Table S1): 47,192 (42.7%) known transcripts from known genes, 57,908 (52.3%) novel transcripts from known genes, and 5511 (5%) novel transcripts from novel genes. The isoforms compared to the reference genome were further clustered to remove redundancy, resulting in 63,419 isoforms, which were classified into 3 types (Figure 3D, Table 2): 18,890 (29.8%) known isoforms (known genes), 2112 (3.3%) novel isoforms, and 42,417 (66.9%) new isoforms. In summary, a total of 2112 novel isoforms and 42,417 new isoforms were identified by SMRT sequencing.

### 2.3. Functional Annotation of Full-Length Transcriptome of H. armiger

**Figure 4:**
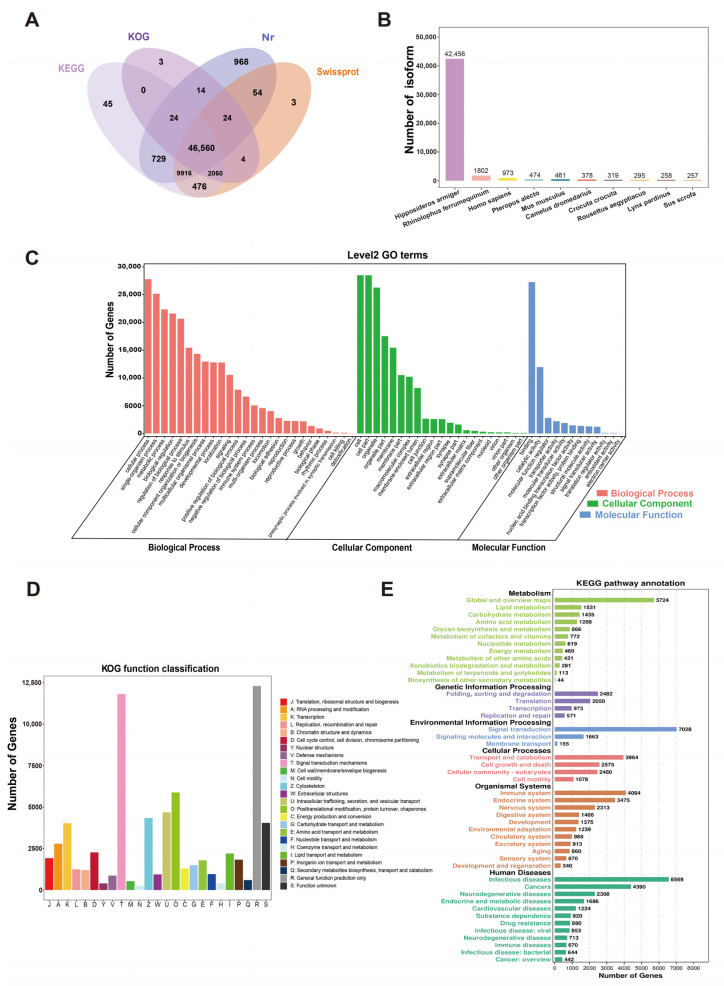
Function annotation of all isoforms. (**A**) Venn diagram of annotations in KEGG, KOG, NR, and SwissProt databases. (**B**) Nr Homologous species distribution diagram of all isoforms. (**C**) Distribution of GO terms for all annotated isoforms in biological process, cellular component, and molecular function. (**D**) KOG enrichment of all isoforms. (**E**) KEGG pathways enrichment of all isoforms.

The number of all mapped isoforms was 63,419. A total of 60,880 isoforms were successfully annotated in the Nr, KOG, KEGG, and Swissprot databases, and 45,460 genes were annotated in four databases, with an annotation rate of 96.00% (Figure 4A). We compared isoforms with NR database for homologous species, and the results showed that the five most abundant isoforms were distributed in *Hipposideros armiger* (42,456), *Rhinolophus ferrumequinum* (1802), *Homo sapiens* (973), *Pteropus alecto* (474), and *Mus musculus* (461) (Figure 4B), suggesting that the most of the isoforms were distributed in *H. armiger* itself, *R. ferrumequinum*, the species closely related to *H. armiger*, as well as the more studied and better quality genomes of *H. sapiens* and *M. musculus*.

Subsequently, GO, KOG, and KEGG functional annotations were performed. The GO functional annotation analysis showed that in the category of biological process, metabolic process (GO:0008152), regulation of biological process (GO:0050789), and response to stimulus (GO:0050896) were the most representative. We found that several terms related to cell and neuronal synapses, including cell (GO:0005623), cell part cellular component (GO:0044464), synapse (GO:0045202), and synapse part (GO:0044456), were significantly enriched in the cellular component category. In the molecular function category, transporter activity (GO:0005215), signal transducer activity (GO:0004871), and translation regulator activity (GO:0045182) were significantly enriched (Figure 4C). We found that the categories of physiological processes associated with auditory nerves were significantly enriched and may play a role in the high-frequency auditory and echolocation abilities of bats. KOG analysis showed that 427 isoforms were assigned to 25 functional clusters, with “general function prediction only”, “Signal transduction mechanisms”, and “Posttranslational modification, protein turnover, chaperones” were ranked as the top three largest categories (Figure 4D).

The KEGG results demonstrated that the isoforms were mapped to 6 main categories with 352 pathways, namely Human Diseases (21,299, 28.70%), Organismal Systems (17,673, 23.81%), Metabolism (13,554, 18.26%), Cellular Processes (10,077, 13.58%), Genetic Information Processing (6076, 8.19%), and Environmental Information Processing (5546, 7.47%). Signal transduction (7028, 9.47%) was the largest group of isoforms annotated, followed by Infectious diseases (6569, 8.85%) and Global and overview maps (5724, 7.71%). In addition, Cancers (4390, 5.91%) and Immune system (4094, 5.52%) also had high annotated expression (Figure 4E).

**Figure 5:**
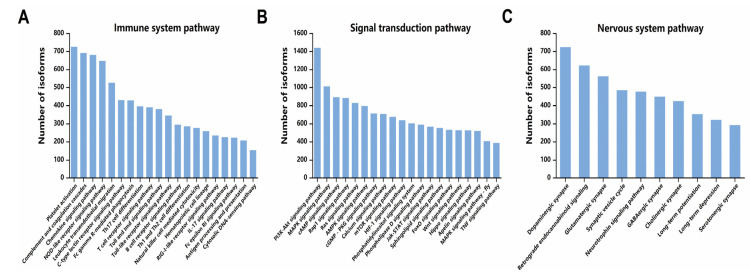
Participation of isoforms in various pathways. (**A**) The proportion of isoforms annotated in the Immune system pathway. (**B**) The proportion of isoforms annotated in the signal transduction pathway. (**C**) The proportion of isoforms annotated in the nervous system pathway.

Next, we selected Immune system pathway, Signal transduction pathway, and Nervous system pathway for further analysis based on the extent of involvement of isoforms in each of the KEGG secondary pathways. We collated the pathways with higher annotated expression in them (Figure 5A–C). Among the Immune system pathways, the most significantly enriched categories were Platelet activation (ko04611), Complement and coagulation cascades (ko04610), and Chemokine signaling pathway (ko04062). In signal transduction pathways, the most significantly enriched pathways were PI3K-Akt signaling pathway (ko04151), MAPK signaling pathway (ko04010), and cAMP signaling pathway (ko04024). In the nervous system pathway, the categories of physiological processes correlated with the auditory nervous system were significantly enriched, such as Dopaminergic synapse (ko04728), Retrograde endocannabinoid signaling (ko04723), Neurotrophin signaling pathway (ko04722), Glutamatergic synapse Glutamatergic synapse (ko04724), and Synaptic vesicle cycle (ko04721).

### 2.4. Prediction and Functional Annotation of Novel Genes

**Figure 6:**
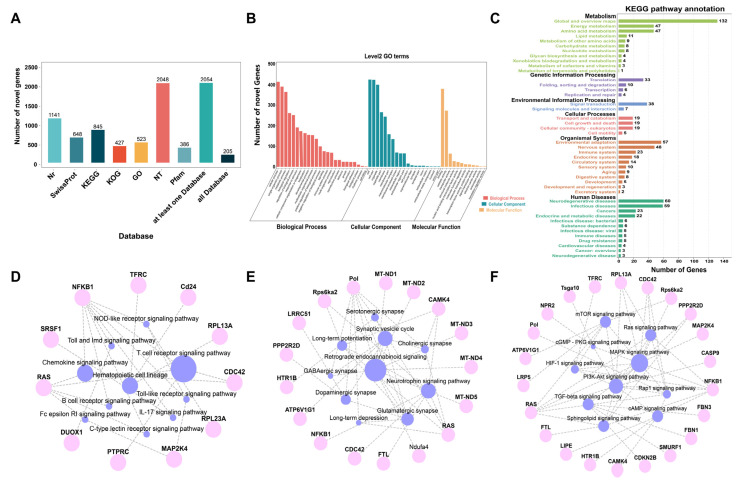
Basic annotation information of novel genes. (**A**) Function annotation of novel genes in all databases. (**B**) Distribution of GO terms for all annotated novel genes in biological process, cellular component, and molecular function. (**C**) KEGG pathways of novel genes. (**D**) Network diagram of Immune system pathways and genes. (**E**) Network diagram of Nervous system pathways and genes. (**F**) Network diagram of Signal transduction pathways and genes.

The 2112 novel genes identified by SMRT sequencing were functionally annotated using the Nr (1141), Swissprot (648), KEGG (845), KOG (427), GO (523), Nt (2048), and Pfam (386) databases. A total of 6018 novel genes were successfully annotated with 205 genes having hits in each database, 457 genes having hits in at least one database, and 2054 genes having hits in at least one database (Figure 6A). By using all annotated novel genes in the *H. armiger* as background, GO analysis showed that “Cell”, “binding”, and “cellular process” were ranked as the most significantly enriched in the “cellular components”, “molecular functions”, and “biological process” categories, respectively (Figure 6B). The KEGG results showed that novel genes were mapped to 236 KEGG pathways, the highest association with several key secondary pathways, including Global and overview maps, Neurodegenerative diseases, Environmental adaptation, Signal transduction, Nervous system and Immune system, etc. (Figure 6C).

The immune system pathway, nervous system pathway, and signal transduction pathway were selected for further analysis based on the extent of involvement of novel genes in each of pathways. Network diagrams of the pathways and genes associations were made (Figure 6D–F). For the tertiary pathways under the immune system, hematopoietic cell lineage (ko04640), NOD-like receptor signaling (ko04621), and T cell receptor signaling (ko04660) are the pathways with the highest number of genes expressed, and *NFKB1* and *RAS* are the most associated genes (Figure 6D). Among nervous system pathways, the pathways with the highest number of genes expressed are retrograde endocannabinoid signaling (ko04723), neurotrophin signaling pathway (ko04722), and synaptic vesicle cycle (ko04721), with *Pol*, *RAS*, and *CAMK4* being the most associated genes (Figure 6E). In the signal transduction pathway, *RAS*, *NFKB1*, *CDC42*, and *RPL13A* were associated with MAPK signaling pathway (ko04010), PI3K-Akt signaling pathway (ko04151), and Ras signaling pathway (ko04014) (Figure 6F).

### 2.5. Structure Analysis

#### 2.5.1. Prediction of Coding Sequences

**Figure 7:**
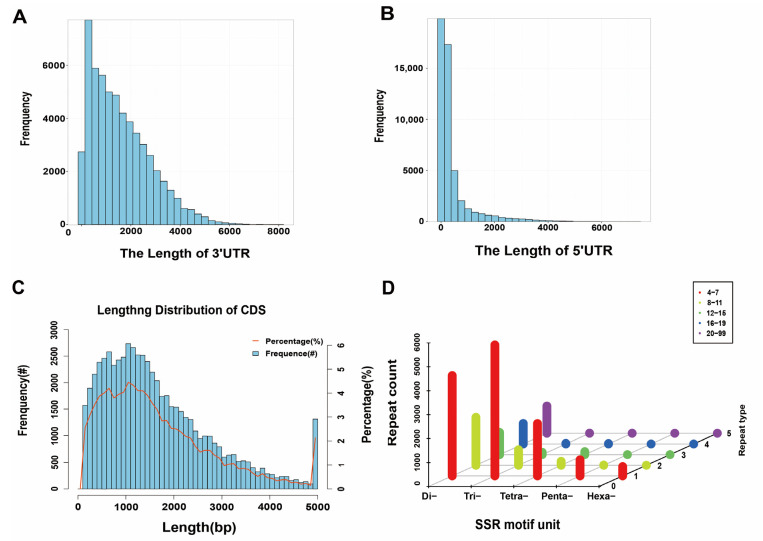
Prediction of the coding sequences and SSR types. (**A**) The length of 3′ UTR. (**B**) The length of 5′ UTR. (**C**) Length distribution of CDSs. (**D**) Distribution map of SSR types.

We identified the number and length of 5′ UTR, 3′ UTR, and CDS by ANGEL software. A total of 61,433 coding sequences with an average length of 1667 bp were predicted, of which 55,808 coding sequences containing start and stop codons were defined as complete open reading frames (ORF). Subsequently, we analyzed the number, size, and length distribution of 5′ UTR and 3′ UTR. In total, 43,165 isoforms were annotated to the 5′ UTR region and 43,557 isoforms were annotated to the 3′ UTR region. The 3′ UTR and 5′ UTR length distributions are shown in Figure 7A,B. The majority of CDSs (42,370 isoforms) (68.97%) were less than 2000 bp in length, 29.06% ranged between 2000 and 5000 bp (17,854 isoforms), and only 1208 isoforms (1.97%) were longer than 5000 bp (Figure 7C). In addition, the number and length distribution of the corrected 5′ UTR and 3′ UTR showed that the genome contained 43,165 5′ UTR and 43,557 3′ UTR, resulting in a total of 116,011 isoforms with an average length of 3443.03 bp. A comparison table of the data before and after correction is shown in Table 3.

#### 2.5.2. SSR Analysis

SSR (Simple Sequence Repeats, Microsatellite) analysis of the transcriptome using MISA 1.0 software revealed 18,919 SSRs. Further analysis indicated that Di-nucleotide repeats (9101) accounted for 48.11%, Trinucleotide repeats (6243), and Tetra-nucleotide repeats (2498) accounted for 33.00% and 13.20%, respectively. However, Penta-nucleotide repeats and Hexa-nucleotide repeats accounted for a small proportion, which were 3.63% and 2.06%, respectively. In addition, several SSRs had repeat numbers of 4–7 and 8–11 (Figure 7D).

#### 2.5.3. Characterization and Analysis of AS and APA Events

**Figure 8:**
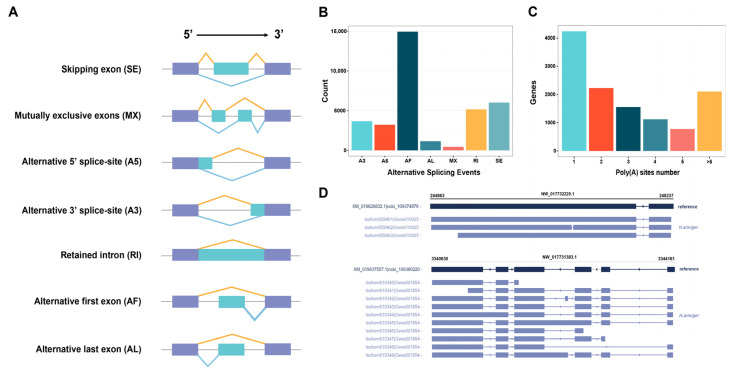
AS and APA analysis of *H. armiger* full-length transcriptome. (**A**) Seven major AS events. (**B**) Number and categories of the AS events identified. (**C**) Distribution of the number of poly (A) sites per gene. (**D**) Two examples for the isoforms generated by the same gene (XM_019629832.1 (*TIGD2*) and XM_019637557.1 (*ACTB*), respectively). The transcripts in the reference genome of *H. armiger* and PacBio were respectively marked with dark blue and purple color. The gray horizontal line and the text above showed the chromosome ID, start, and end position of the terminal gene region.

AS is significant for regulating gene expression and generating protein diversity, which can increase structural and functional polymorphism of transcripts and proteins. In the study, we identified 34,611 AS events in *H. armiger* based on PacBio sequencing results. To obtain more detailed information, we calculated the frequencies of seven major AS events, including A3, A5, AF, AL, MX, RI, and SE (Figure 8A). The number of Alternative First Exons (14,941, 43.17%), Skipping Exon (6005, 17.35%), and Retained Intron (5174, 14.95%) was much higher than the other four AS events. For example, Alternative 3′ Splice Sites (3664, 10.59%), Alternative 5′ Splice Sites (3221, 9.31%), Alternative Last Exons (1158, 3.35%), and Mutually Exclusive Exons (448, 1.29%) (Figure 8B). Furthermore, 8797 genes identified by *H. armiger*, 3.43% had only one isoform, 96.57% had two or more isoforms, and the novel gene coding for ncbi_109388401 (*SLC38A2*) had the most splice variants with up to 52. Two examples of *H. armiger* AS events were shown in Figure 8D, ncbi_109374879 (*TIGD2*) was annotated with an isoform (XM_019629832.1) in the reference genome, while three isoforms were identified using our PacBio data; ncbi_109380220 (*ACTB*) was annotated with an isoform (XM_019637557.1) in the *H. armiger*, and as many as 10 isoforms were identified based on the full-length transcriptome.

PacBio sequencing also promotes the study of APA sites, and we examined the 3′ end of the isoforms sequenced by PacBio SMRT to identify the APA sites of the genes accurately in *H. armiger*. A total of 66,010 poly (A) sites were identified in 12,029 genes identified, 4237 genes showed one poly (A) sites, and 2105 genes contained at least five poly (A) sites. On average, each gene contained 1.04 poly (A) sites (Figure 8C).

#### 2.5.4. LncRNA and TF Identification

**Figure 9:**
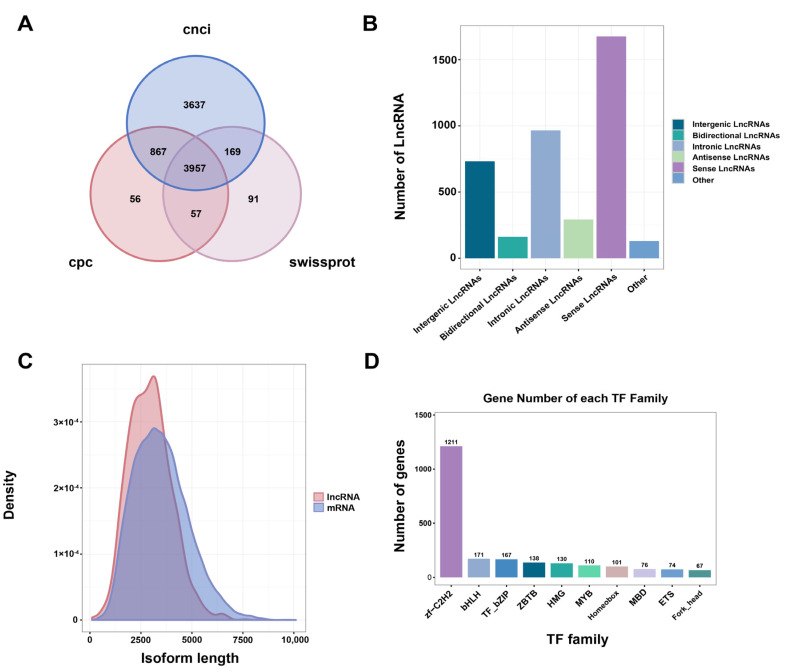
The identification of lncRNA and TFs based on the SMRT sequencing in *H. armiger*. (**A**) Venn diagram of lncRNA predicted by CNCI, CPC, and Swissprot tools. (**B**) Proportions of different types of lncRNAs. (**C**) Length distribution of identified lncRNAs and mRNA in *H. armiger*. (**D**) The number and family of TFs were predicted by SMRT.

LncRNAs play an essential role in regulating gene expression in most eukaryotes. We identified 86,304,937, and 4274 isoforms by CNCI, CPC, and Swissprot, respectively. Combining the results of three methods, a total of 3957 lncRNAs were predicted, including 2542 new lncRNAs (64.2%) and 1415 novel lncRNAs (35.8%) (Figure 9A). In addition, we classified new LncRNAs into five major categories based on their position on the genome relative to protein-coding genes: Intergenic LncRNAs, Bidirectional LncRNAs, Intronic LncRNAs, Antisense LncRNAs, and Sense overlapping LncRNAs (Figure 9B). Comparing the density of isoforms length distribution of lncRNAs and original mRNAs, it was found that more lncRNAs between 2500 nt and 3500 nt in length than mRNAs, with the longest predicted lncRNA not exceeding 10,000 nt (Figure 9C).

Transcription factors (TFs) play an important role in regulating animal growth and development. In this study, they were identified and classified using the animalTFDB 2.0 database. A total of 3150 TFs from 63 families were identified, and the top 10 TF families were zf-C2H2, bHLH, TF_bZIP, ZBTB, HIMG, MYB, Homeobox, MBD, ETS, and Fork_head. Among them, zf-C2H2 family (1211, 38.44%) was the most representative, followed by the bHLH family (171, 5.43%) (Figure 9D).

### 2.6. Gene Structure Optimization

**Figure 10:**
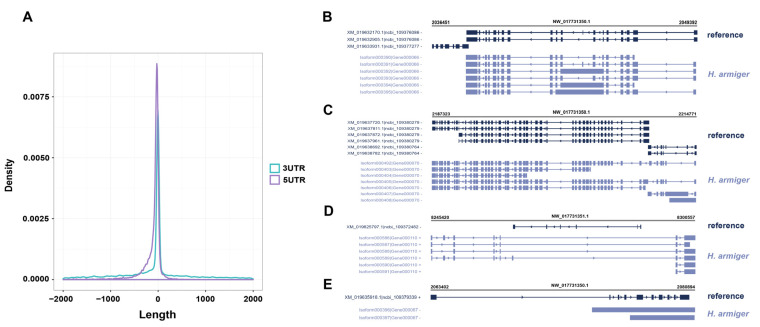
Optimization of *H. armiger* gene structure. (**A**) UTR length variation frequency distribution. (**B**,**C**) Isoforms spanning two or more reference genes. (**D**) An example classified as intron isoforms. (**E**) An example classified as Exonic overlap isoforms.

To optimize the gene structure of the *H. armiger* reference genome, each isoform was compared with the existing gene model annotated by ncbi_GCF_001890085.1. We performed statistical analysis of 5′ UTR and 3′ UTR lengthening or shortening for isoforms classified as known. The frequency distribution of UTR length changes is shown in Figure 10A. Then, we compared the isoforms classified as new with the reference genes, the isoforms in Figure 10B,C (Isoform000390, Isoform000391, Isoform000392, Isoform000393, Isoform000394, Isoform000395 and Isoform000402, Isoform000403, Isoform000404, Isoform000405, Isoform000406, Isoform000407, Isoform000408) span two or more reference genes (XM_019632170.1 (*NRBP1*), XM_019632905.1 (*NRBP1*), XM_019633931.1 (*KRTCAP3*) and XM_019637720.1 (*CAD*), XM_019637811.1 (*CAD*), XM_019637872.1 (*CAD*), XM_019637961.1 (*CAD*), XM_019638692.1 (*ATRAID*), and XM_019638782.1 (*ATRAID*)). These isoforms are known as Span isoforms.

Finally, we analyzed the isoforms classified as novel compared to the reference genes, three types of isoforms were detected. The numbers and categories were 202 Intron isoforms, 536 Exonic overlap isoforms, and 1374 other novel isoforms, which are collectively referred to as antisense isoforms. As shown in Figure 10D, an example of Intron isoforms, isoforms (Isoform000586, Isoform000587, Isoform000588, Isoform000589, Isoform000590, Isoform000591) was located inside the intron of the reference gene (XM_019625797.1, *MYOZ2*). Figure 10E are Exonic overlap isoforms, and isoforms (Isoform000396, Isoform000397) and the reference gene (XM_019635918.1, *PPM1G*) are located on different strands and have exon overlap between them.

## 3. Discussion

Previously, the genome (ncbi_GCF_001890085.1) of *H. armiger* was reported by using the Next-generation genome sequencing technology (NGS) [17], the predicted genome size was 2.18 Gb, and a total of 476.5 Gb sequences and 827,722 single nucleotide polymorphisms (SNPs) were identified. The previous genome dataset of *H. armiger* provided a valuable starting point for further research and can help researchers understand the genetic bases of the species. Although the previous reference genome constitutes the latest *H. armiger* gene annotation, these sequences rarely cover the entire transcripts. The transcripts obtained in this study are almost more than twice the number of transcripts recorded in the annotation of the *H. armiger* reference genome. Mapping full-length transcriptome data into the genome is highly effective method for accurately detecting gene structure [28]. Data from multiple tissues and organs in the body of species can improve the discovery of almost all genes in the genome. Still, the limitations of sequencing technology often force researchers to trade-off between sampling depth and completeness [28,29].

In the last decade, with the development of molecular biology and continuous improvement of technological tools, next-generation sequencing (NGS) has been widely used for gene discovery and research [30,31,32]. RNA-seq has become a necessary technical means for assessing whole RNA expression patterns [33]. However, NGS sequencing fragment lengths are typically short in read length, which is inadequate for accurate reconstruction and reliable expression estimation of transcript variants, especially for low-quality genomes or non-model species without a reference genome [25]. The annotation of reference genomes is often not accurate enough due to the limitations of short-read sequencing. In contrast, the Full-length cDNA assembled by PacBio SMRT sequencing technology is a good standard for gene annotation. Compared with the previous next-generation sequencing technologies, the most prominent feature is single molecule real-time sequencing, which eliminates the need for PCR amplification, reducing the cost and sequencing time. It implements the DNA polymerase’s continuity and reaction speed, and high precision directly measuring RNA sequences, has long reads [34], and identifies the complete structure of individual transcript, which reveals the complexity of transcriptome, compensates for the shortcomings of NGS technology, and optimizes the original species genome annotation, with annotation for more transcripts.

Previous studies on bat genetics and genomics were mainly based on short-read sequencing (NGS) technologies, which might no longer meet the need for growing researches. For instance, Wang et al. (2018) [35] performed a comparative transcriptome analysis of three bat cochlear species using RNA-Seq data by using NGS to evaluate the changes in gene expression among the three echolocating bats. An overview of the genetic basis of differences between echolocating bats reveals different nervous system activity during auditory perception in the cochlea, especially in CF bats. Moreno-Santillán et al. (2019) [36] generated complete transcriptomes from the liver tissue of five different families of tropical bats by using NGS, which is the first cross-species comparison of transcriptome and gene expression in tropical bats. Zhao et al. (2019) [37] performed transcriptome sequencing analysis of *Rhinolophus ferrumequinum* with different acoustic phenotypes by using NGS. The discovery of variability in gene expression and sequence differences at the molecular level may help to provide evidence to elucidate the genetic basis of geographical variation in echolocation signals in *R. ferrumequinum*. Even so, the NGS can no longer be sufficient for in-depth study of species, and high quality genomic data are necessary in future studies.

Therefore, more abundant and complete transcripts are essential for better research in the field of bat acoustics and the adaptive evolution of specific traits. SMRT sequencing based on Pacbio can directly read cDNA and obtain high-quality, complete, and accurate reads, providing more detailed and comprehensive data characteristics at the molecular level [38]. Whereas, to our knowledge, there is only one study that has applied PacBio SMRT sequencing to bat species. Ma et al. (2020) [39] used PacBio sequencing technology to identify two subspecies (*R. a. hainanus* and *R. a. himalayanus*) of the constant-frequency (CF) bat *Rhinolophus affinis* and the frequency-modulated (FM) bat *Myotis ricketti* for the first time. It is the first time that PacBio sequencing technology has been used for bat species. The main content was to investigate the genetic basis of intraspecific echolocation call frequency variation. Overall, 3,444,947, 3,255,638 and 3,403,451 subreads were obtained for the three bats, with total base numbers of 6,448,987,299 bp, 6,504,282,447 bp, and 7,190,237,257 bp, with average lengths of 1872 bp, 1998 bp, and 2113 bp, respectively. However, the researchers only studied for cochlea, which was not enough to summarize the complete genome of the bat species. No further follow-up comparative analysis of these data was performed. In this study, a total of 40,724,724 subreads were obtained with total base number of 125,546,653,021 bp and an average length of 3082 bp. By comparing with the above studies, the results of full-length sequencing based on NGS genome annotation have achieved good performance in terms of sequence quality and gene number, and abundant transcripts were also identified.

To identify as many transcripts as possible, we improved the current genetic model for *H. armiger* genome annotation by mixing RNA samples from five organs (cochlea, brain, heart, liver, and muscle) for PacBio SMRT sequencing. Our results provided the first full-length transcriptome resource for *H. armiger*. About 120 Gb of subreads data were obtained and 1,630,747 CCSs were generated, of which 1,472,058 were identified as Full-length Non-chimeric (FLNC) reads, accounting for 90.27% of all CCSs. A total of 91,477 high-quality isoforms and 3504 low-quality isoforms were obtained after polishing and 94,981 consensus isoforms were obtained after de-redundancy of the sequences. High-quality isoforms were mapped to the reference genome of *H. armiger* (ncbi_GCF_001890085.1) with a mapping rate of 89.5%, indicating that the sequencing data were of high quality. The isoforms compared to the reference genome were further clustered to remove redundancy, resulting in 63,419 isoforms, comprising 18,890 (29.8%) known isoforms (known genes), 2112 (3.3%) novel isoforms (novel genes), and 42,417 (66.9%) new isoforms (known genes). These data not only enrich the transcriptional information of the *H. armiger* draft genome sequence but could also be applied for functional studies of key genes in *H. armiger* in the future.

### 3.1. Prediction and Functional Annotation of Optimized Isoforms

We performed functional annotation on the isoforms optimized and aligned to the genome after clustering redundancy removal. A total of 60,880 isoforms were successfully annotated in NR, KOG, KEGG, and Swissprot databases. Except for the species studied here, *H. armiger* and *R. ferrumequinum* had the highest homology of annotated sequences, which reflects the high affinity between *H. armiger* and *R. ferrumequinum*. We speculate that they belong to Rhinolophoidea subfamily of the Yinpterochiroptera suborder, which would provide valuable data for a detailed comparison of gene expression between the two species in the future. Previous genome sequencing studies have shown that most of the genes that specify key biological functions were shared by all eukaryotes [40]. In this study, KOG database annotation showed that most of the transcripts were enriched in General function prediction only and Signal transduction mechanisms. GO and KEGG enrichment analysis showed that synapse synapses (GO:0045202), synapse part (GO:0044456), transporter activity (GO:0005215), signal transducer activity (GO:0004871), and translation regulator activity (GO:0045182) were significantly enriched in the category of physiological processes for hearing, while the Dopaminergic synapse (ko04728), Retrograde endocannabinoid signaling (ko04723), Neurotrophin signaling pathway (ko04722), and Glutamatergic synapse (ko04724) in the nervous system pathway of in KEGG may also be involved in the intrinsic molecular regulation of high-frequency auditory activity in CF bats.

### 3.2. Identification of Novel Genes Potential Related with Immune, Nervous and Signal Transduction

In addition, GO and KEGG functional annotation were performed on 2,112 novel genes detected in this study that were not annotated in the original genome. We found several important genes that may be involved in regulating the immune and nervous system in bats, such as *RAS*, *NFKB1*, *Pol*, and *CAMK4*, which highly correlates with Neurotrophin signaling pathway (ko04722), Synaptic vesicle cycle (ko04721), Retrograde endocannabinoid signaling (ko04723), NOD-like receptor signaling (ko04621), and T cell receptor signaling (ko04660). Wang et al. (2018) [35] conducted a comparative transcriptome study on the cochlea of CF and FM bats and found that the up-regulated genes in CF bats were significantly enriched. Through differential analysis, the terms closely related to various components of the auditory nervous system were screened out, and a large number of neuron and synapse-related categories were found, including neuronal parts, neuronal projections, synapses, synapses parts, membrane and synaptic membrane, indicating significant differences in neural activity in the cochlea of CF bats compared to other bats, as well as essential and unusual synaptic transduction. These entries are also broadly consistent with our current findings. Overall, the transcriptome data obtained in this study can be used as a standard reference and database in conjunction with previous studies when studying echolocating bats and evolution of species in the future.

### 3.3. Gene Structures Increased the Complexity and Diversity of Transcripts

In eukaryotes, transcripts are highly complex and diverse as precursor mRNAs are subject to multiple post-transcriptional modification processes, such as AS and APA [41,42]. It has been reported that RI and SE are the primary forms of AS expression in eukaryotes, where SE is higher in animals and RI is common in all eukaryotes, including plants [43,44]. In *H. armiger*, 34,611 AS events were identified by PacBio sequencing. Apart from AF, SE and RI were the most common AS types. At present, there is still a lack of more specific data for AS research on bat species based on PacBio long-read sequencing, which will be one of the priorities for future studies on bats. Previous studies have shown that APA is indispensable for RNA transport, localization, stability, and translation processes, and APA of RNA affects gene function by gene expression and altering the complexity of the transcriptome [45,46]. Our study also provides a comprehensive genome-wide APA map in which 66,010 poly (A) sites were identified for 12,029 genes and one poly (A) site was shown for 4237 genes. A total of 2105 genes contained at least five poly (A) sites, with an average of 1.04 poly (A) sites per gene. In conclusion, AS and APA transcripts were identified in this study, which provided further evidence of the complexity of the *H. armiger* transcriptome, improving the transcript information significantly in the original *H. armiger* genome annotation, as well as providing valuable information for studies of other bats in relation to AS and APA.

SSRs were widely used in genetic diversity detection, genetic map construction, gene expression regulation, and other fields [47]. A total of 18,919 SSRs were found by analyzing the SSRs of *H. armiger* transcriptome. The SSRs obtained in this study could be used for developing microsatellite markers for individual identification and provided a reference for the population genetic polymorphism of *H. armiger*. PacBio SMRT sequencing offered advantages in mining lncRNAs at the transcriptome level and can be used to explore lncRNAs in certain plants and animals [48,49]. In our study, 1415 novel lncRNAs were identified based on PacBio sequencing data. These newly identified lncRNAs will provide additional valid candidates for future functional characterization. Transcription factors (TFs) play a crucial part in the regulation of gene expression. Their characteristics may provide important clues to understanding the regulatory mechanism of gene expression [50]. We identified 3150 putative TFs from 63 families, of which the zf-C2H2 family was the most representative, followed by the bHLH family. zf-C2H2 has functions such as recognizing and binding of specific DNA fragments involved in the regulation of gene expression. BHLH constitutes a large family of eukaryotic proteins, which is the most extensive class of eukaryotes. They can participate in various processes in cells and play a vital role in the regulation of biological growth and development [51], which may affect the synthesis of various bioactive substances indirectly.

### 3.4. PacBio Sequencing Optimized Genome Annotation

The full-length transcriptome sequencing can modify the genomic information of species to a certain extent. The 110,611 isoforms described here are almost more than twice the number of transcripts recorded in the annotation of the *H. armiger* reference genome. Although most of the PacBio isoforms were novel isoforms from known genes, 4.98% of these isoforms provided evidence for 2112 novel genes. As evidence that full-length transcriptome data can also correct or improve the majority of misannotated gene models, we present two *H. armiger* genome reference genes, which were annotated with only one transcript in the reference genome but were annotated with more than 3 or 10 isoforms in Pacbio sequencing. Our analysis suggests that the new transcriptome data have great potential to improve the current annotation of the *H. armiger* genome. In summary, our sequencing results optimized and replenished the current *H. armiger* genome data, which offer new information about isoforms and transcripts. We believe that in the future, as the omics analysis technology continues to develop, that the genome annotation of *H. armiger* will also be constantly updated, thus helping researchers to carry out extensive research.

## 4. Materials and Methods

### 4.1. Sample Collection and RNA Preparation

Two adult male *H. armiger* weighing 66.0 ± 1.0 g were collected on 5 July 2021 in Fei Long Cave, Xingyi, Guizhou Province, China (24°58′ N, 104°52′ E), to obtain comprehensive transcript information. The bilateral cochlea, brain, heart, liver, and muscle tissues were collected from both individuals and snap-frozen immediately in liquid nitrogen overnight. Then, those tissues were stored in an ultra-low temperature freezer at −80 °C until RNA extraction.

### 4.2. Library Construction and Single-Molecule Real-Time (SMRT) Sequencing

Total RNA was extracted by grinding five kinds of tissues from two individuals in TRIzol reagent (Life Technologies, Carlsbad, CA, USA) on dry ice and processed following the protocol provided by the manufacturer. Five kinds of tissues from two individuals were pooled to extract total RNA for library construction and sequencing. The integrity of the RNA was determined with the Agilent 2100 Bioanalyzer and agarose gel electrophoresis. The purity and concentration of the RNA were determined with the Nanodrop micro-spectrophotometer (Thermo Fisher, Waltham, MA, USA). The RNA Integrity Number (RIN) was tested at 7.9 and the concentration was 425 ng/μL.

Then, the mRNA was enriched by using Oligo (dT) magnetic beads and the enriched mRNA was reverse-transcribed into cDNA using the Clontech SMARTer PCR cDNA Synthesis Kit (Takara, Shiga, Japan). Optimization was used to determine the optimal number of amplification cycles for downstream large-scale PCR reactions, and the optimized number of cycles were used to generate double-stranded cDNA. In addition, cDNAs larger than 4 kb were selected using the BluePippinTM Size-Selection System and mixed evenly with equal amounts of cDNAs that were not size-selected. Large-scale PCR was performed on libraries of different sizes to be used in the construction of the following SMRT-bell libraries, which were used for DNA damage repair, end repair, and ligation to sequencing adapters. Finally, SMRTbell templates were annealed to sequencing primers and bound to polymerase and sequenced on the PacBio Sequel platform, using P6-C4 chemistry with 10 h movies.

### 4.3. PacBio Sequencing Processing

Raw sequencing reads of cDNA libraries were classified for clustering into consistent transcripts using the Pacific Biosciences SMRT Link v5.0.1 pipeline [22]. Circular Consensus Sequence (CCS) reads were extracted from subreads BAM files with a minimum total pass rate of 1 and a minimum read score of 0.65. Following that, CCS fragments were classified into full-length non-chimeric (FL), non-full-length (nFL), chimeras and short reads based on cDNA primers and polyA tail signal, and reads shorter than 50 bp were discarded. Subsequently, full-length non-nested (FLNC) reads were clustered by Iterative Clustering Error correction (ICE) software to generate the cluster consensus isoforms. To improve the accuracy of PacBio reads, the above-obtained cluster consensus isoforms were polished by Quiver software to obtain FL polished high quality consensus sequences (accuracy ≥ 99%).

### 4.4. Mapping to the Reference Genome

The corrected high quality consensus sequences were mapped to the reference genome of *H. armiger* (ncbi_GCF_001890085.1) using GMAP [52] and redundant transcripts were collapsed with minimum identity of 95% and minimum coverage of 99%. The final isoforms obtained were compared with the reference genome annotation and classified into three types: known isoforms, novel isoforms, and new isoforms. Consistent sequences that align to unannotated regions of genes are considered novel isoforms, and consistent sequences that align to different exons of a known isoform are considered new isoforms.

### 4.5. Functional Annotation of Isoforms and Novel Genes

To investigate the function of isoforms, according to the NCBI non-redundancy protein (Nr) database (http://www.ncbi.nlm.nih.gov, accessed on 15 November 2021) and eukaryotes homology cluster (KOG) database Protein data bank (http://www.ncbi.nlm.nih.gov/KOG, accessed on 20 November 2021), Switzerland (SwissProt) (http://www.expasy.ch/sprot, accessed on 20 November 2021) and the Kyoto encyclopedia (KEGG) gene and genome database (http://www.genome.jp/kegg, accessed on 22 November 2021), new isoforms were BLAST analyzed against using BLASTx program (http://www.ncbi.nlm.nih.gov/BLAST/, accessed on 22 November 2021) at an E-value threshold of 1 × 10^−5^ to evaluate sequence similarity with genes of other species. The Nr annotation results of isoforms were annotated with gene ontology (GO) using Blast2GO software [53]. In the analysis, the top 20 highest scoring isoforms with no less than 33 HSP (High-scoring Segment Pair) hits were selected for Blast2GO analysis. Following that, isoforms were functionally classified using WEGO software (version 1.0) [54]. Herein, we defined novel (compared to NCBI gene-build) isoforms as genes putatively encoding detected isoforms that do not match any annotated genes in the *H. armiger* reference genome (ncbi_GCF_001890085.1). GO and KEGG annotation methods of the novel genes were the same as above.

### 4.6. Analysis of AS and APA

One of the most significant advantages of PacBio sequencing is its ability to identify AS and APA by comparing different isoforms of the same gene. In this study, the SUPPA [55] tool was used to predict variable splicing and seven major types were detected, including Alternative 5′ Splice Sites (A5), Alternative 3′ Splice Sites (A3), Alternative First Exons (AF), Alternative Last Exons (AL), Mutually Exclusive Exons (MX), Retained Intron (RI), Skipping Exon (SE), and were classified and counted. The analysis of poly(A) was carried out using all high-quality isoforms aligning to transcripts and the end position is the potential alternative polyadenylation (APA) sites. MEME analysis was conducted on the sequence of 50 nucleotides upstream of the total poly-A sites.

### 4.7. Simple Sequence Repeats (SSR) Prediction

Microsatellites (MISA, http://pgrc.ipk-gatersleben.de/misa/, accessed on 9 December 2021), also known as simple sequence repeats (SSR), are uniformly distributed in eukaryotic genomes. Microsatellite markers are composed of tandem repeat segments of 2 to 6 nucleotides. Owing to the number of repeat units being highly variable between individuals and abundant, microsatellite markers are widely used. In this study, the minimum number of repeats in the identification process varies depending on the difference in the unit size of the repeats, with the minimum number of repeats being 2–6, 3–5, 4–4, 5–4, and 6–4. An SSR is if the distance between two SSRs is less than 100 bp. SSRs are classified into five types: Di-, Tri-, Tetra-, Penta- and Hexa-.

### 4.8. Prediction and Analysis of ORF, LncRNA, and TFs

The open reading frame (ORF) of all isoforms was predicted using ANGEL [56] software to obtain coding sequences (CDS), protein sequencesl and untranslated region (UTR) sequences. Both CNCI (version 2) [57] and CPC [58] (http://cpc.cbi.pku.edu.cn/, accessed on 9 December 2021) were used to assess the protein-coding potential of novel isoforms and new isoforms under default parameters. Meanwhile, isoforms were mapped to the SwissProt database to evaluate protein annotations. The intersection of non-protein-coding potential results and non-protein annotation results was chosen as the lncRNAs. To better annotate lncRNAs at the evolutionary level, Infernal [59] (http://eddylab.org/infernal/, accessed on 9 December 2021) was used for sequence alignment. LncRNAs were classified according to secondary structure and sequence conservation. For transcription factor (TF) analysis, TF families were predicted by matching the protein-coding sequences of isoforms to the TFdb (http://www.bioguo.org/AnimalTFDB/, accessed on 12 December 2021) of animals using hmmscan software (version 3.0).

### 4.9. Gene Structure Optimization

To optimize the gene structure of the *H. armiger* reference genome, each isoform was compared with the existing gene model annotated by ncbi_GCF_001890085.1. Known isoforms are the lengthening or shortening of the 5′ UTR or 3′ UTR, and other isoforms were further divided into span isoforms, exonic overlap isoforms, and intron isoforms span isoforms based on their exon structure (splicing junctions).

## 5. Conclusions

We analyzed the full-length transcriptome of *H. armiger* with PacBio SMRT sequencing for the first time. AS and APA analysis, CDS prediction, transcription factors, SSR discovery, and lncRNA prediction were performed under optimized and replenished *H. armiger* genome annotation. A number of novel genes related to nervous perception and immunity were identified, which may be involved in bat nervous and immunity system regulation in echolocation. Our results may provide valuable information for improving the existing gene model in *H. armiger* and demonstrate that long-read sequencing is complementary to short-read sequencing for cataloging and quantifying isoforms. The optimized genome annotation could be jointly analyzed together with multiomics data and used as a reference genome for single-cell sequencing to take insights concerning animal evolution and genetic protection, preferably in the future.

## Figures and Tables

**Table 1:** The PacBio SMRT sequencing information of *H. armiger*.

Categories	Datasets
Number of bases sequenced (bp)	125,546,653,021
Number of subreads	40,724,724
Number of subreads average length	3082
Number of N50 length	3573
Number of CCS bases	5,765,831,264
Number of CCS reads	1,630,747
Number of mean CCS reads length	3535
Number of mean passes	22
Number of full-length non-chimeric reads	1,472,058
Full-length non-chimeric percentage (FLNC%)	90.27%
Number of mean Length of FLNC	3440
Number of consensus isoforms	94,981
Number of polished high-quality isoforms	91,477
Number of polished low-quality isoforms	3504

**Table 2:** Statistical table results of gene expression.

Terms	Number of Reads
All reference gene number	18,949
All reference transcript number	47,179
All mapped gene	12,029
All mapped Isoform	63,419
known isoform	18,890
novel isoform	2112
new isoform	42,417
known transcripts from known genes	47,192
novel transcripts from known genes	57,908
novel transcripts from novel genes	5511

**Table 3:** Gene structure analysis before and after correction.

Type	Transcript	Average Length	3′-UTR	5′-UTR
Before	47,192	3317	43,557	44,526
After	110,611	3343	43,557	43,165

## Data Availability

The raw reads generated for this study can be accessed from NCBI Sequence Read Archive (SRA) under SRR22307099. Other data sets generated in this study will be available from the corresponding author upon request.

## Supplementary Materials

The following supporting information can be downloaded at: https://www.mdpi.com/article/10.3390/ijms24054937/s1.
