# Supplementary material for: Full-Length Transcriptome of the Great Himalayan Leaf-Nosed Bats (Hipposideros armiger) Optimized Genome Annotation and Revealed the Expression of Novel Genes
Source: Int J Mol Sci. 2023 Mar 3;24(5):4937. doi: 10.3390/ijms24054937 (PMC10003721; doi:10.3390/ijms24054937)
Supplement: Supplementary file 1 [file ijms-24-04937-s001.zip › ijms-2202289-supplementary/Table S1.pdf]

Table S1. Statistical table of PacBio sequenced data mapping results.

Terms	Number of reads	Percentage (%)
Total Hq Isoform number	91,477	
Total mapped	81,871	89.50%
multiple mapped	4567	5.09%
Uniquely mapped	77,214	84.41%
Reads map to "+" strands	38,948	42.58%
Reads map to "−" strands	38,266	41.83%
